# Antithrombotic Activity of Heparinoid G2 and Its Derivatives from the Clam *Coelomactra antiquata*

**DOI:** 10.3390/md20010050

**Published:** 2022-01-05

**Authors:** Guanlan Chen, Rui Zeng, Xin Wang, Hongying Cai, Jiajia Chen, Yingxiong Zhong, Saiyi Zhong, Xuejing Jia

**Affiliations:** 1 Guangdong Provincial Key Laboratory of Aquatic Products Processing and Safety, School of Food Science and Technology, Guangdong Ocean University, Zhanjiang 524088, China; cgl202112@163.com (G.C.); zengrray@163.com (R.Z.); w18800465398@163.com (X.W.); 13414866246@163.com (H.C.); 15867125821@163.com (J.C.); zyxsaphi@163.com (Y.Z.); jiaxj@gdou.edu.cn (X.J.); 2 Guangdong Province Engineering Laboratory for Marine Biological Products, School of Food Science and Technology, Guangdong Ocean University, Zhanjiang 524088, China; 3 Shenzhen Institute, Guangdong Ocean University, Shenzhen 518108, China; 4 Collaborative Innovation Center of Seafood Deep Processing, Dalian Polytechnic University, Dalian 116034, China

**Keywords:** clam heparinoid, molecular weight, antithrombotic, oral administration, gut microbiota

## Abstract

Clam heparinoid G2 (60.25 kDa) and its depolymerized derivatives DG1 (24.48 kDa) and DG2 (6.75 kDa) prepared from *Coelomactra antiquata* have been documented to have excellent fibrinolytic and anticoagulant activity. In this study, to further explore the antithrombotic activity of G2, DG1 and DG2, azure A, sheep plasma, and clot lytic rate assays were used to determine their anticoagulant and thrombolytic activity in vitro. The results indicated that the anticoagulant titer of G2 was approximately 70% that of heparin and the thrombolytic activity of DG2 was greater than G2, DG1, and heparin activities. Moreover, in a carrageenan-induced venous thrombosis model, oral administration of G2 and DG1 each at 20 mg/kg and 40 mg/kg for 7 days significantly reduced blacktail thrombus formation, increased tissue-type plasminogen activator, fibrin degradation products, and D-dimer levels, decreased von Willebrand factor and thromboxane B2 levels, and restored phylum and genus abundance changes of intestinal bacteria. DG2 had no antithrombotic effect. At 20 mg/kg, G2, DG1, and heparin had comparable antithrombotic activities, and DG1 at 40 mg/kg had more muscular antithrombotic activity than G2. Thus, DG1 could be an antithrombotic oral agent owing to its more robust antithrombotic activity and lower molecular weight.

## 1. Introduction

Thromboembolic diseases, such as pulmonary embolism, stroke, and myocardial infarction, are characterized by the formation of blood clots within the veins or arteries in different tissues and the travel and lodging of thrombus. They are a global health problem [1]. Mammalian-derived heparin, obtained from porcine intestine and bovine lung, is an anticoagulant drug that prevents and treats thromboembolic disease [2]. Heparin treatment has several side effects, including thrombocytopenia and bleeding, in addition to the risk of contamination with pathogenic agents, which limit its application [3].

Heparinoids are heparin-like molecules, including heparin, heparan sulfate, fucoidans, chitosan, and other sulfated polysaccharides with structural and functional similarities to heparin [4]. Marine-derived heparinoids, which possess potent antithrombotic properties in multiple pathways and few side effects, are promising alternatives to heparin [5,6]. Assaâd et al. extracted a sulfated glycosaminoglycan from European eel skin that exhibited an anticoagulant activity and had negligible hemolytic activity toward human erythrocytes [7]. Cao et al. reported anticoagulant, antiplatelet aggregation, thrombolytic, and antithrombotic activities of a novel sulfated polysaccharide from the marine green alga *Monostroma nitidum* [8]. Racquel et al. reported that polysaccharides from *Geoffroea spinosa* bark have anticoagulant and antithrombotic activities and pose no hemorrhagic risks [9]. In addition, low-molecular-weight heparinoids have received increasing attention owing to their good processability, high bioavailability, and low risk of inducing bleeding [10,11]. The depolymerized fragments (dFCSc) from fucosylated chondroitin sulfate from the sea cucumber *Cucumaria frondosa* exhibited a better antithrombotic-hemorrhagic ratio than native FCSc in an electrically induced arterial thrombosis model in rats [12]. The low-molecular-weight fraction dHG-5 prepared via the partial depolymerization of native fucosylated glycosaminoglycan from the sea cucumber *Holothuria fuscopunctata* represent a source of potential anticoagulants to be explored [13].

The largest organ that digests and absorbs nutrients is the gut, with 10^14^ bacteria and 100 times more genes than the human genome [14]. Several studies have demonstrated that gut flora is associated with thrombotic diseases [15,16,17]. Thrombotic diseases can induce disordered intestinal flora, increase the accumulation of harmful metabolites, and promote the formation of thrombi [18,19,20]. Moreover, regulation of the intestinal flora can slow down thrombotic diseases to an extent [21], as can synergistic antithrombotic drugs [22]. These studies suggest that intestinal flora is a new target of antithrombotic medications [23,24]. In addition, the human intestine lacks enzymes that use sulfated polysaccharides [25], whereas heparin can be used by human intestinal flora and exerts a regulatory effect [26]. However, it remains unclear whether heparin can prevent the occurrence of thrombus, considering its biological activities against gut microbiota imbalance.

The heparinoid G15 from the clam *C. antiquata* is a homogeneous glycosaminoglycan with potent anticoagulant and fibrinolytic activity that is mainly composed of →4)-α-IdoA2S-(1→4)-α-GlcNS3S6S (or GlcNS6S)-(1→4)-β-GlcA-(1→4)-α-GlcNS6S (or GlcNAC)-(1→ [27]. G2 was previously prepared by using the extraction of G15 as a reference [27]. In addition, its low-molecular-weight fragments, DG1 and DG2, are derived from vitamin catalytic free-radical depolymerization. In previous studies, a decrease in the molecular weight of clam heparinoid was accompanied by an increase in fibrinolytic activity. Still, the medium-molecular-weight degradation product DG1 showed more muscular anti-Xa/IIa factor activity than G2 and DG1 [28].

To further explore the antithrombotic activity of G2, DG1, and DG2 in this present study, azure A, sheep plasma, and clot lytic rate assays were used to determine their anticoagulant and thrombolytic activity in vitro. Additionally, a carrageenan-induced thrombosis model was established in this study to compare the antithrombotic activity of clam heparin G2 and its degradation products. Meanwhile, in order to explore the potential mechanisms of clam heparinoids in the treatment of thrombosis, their effects on plasma levels of blood factors related to coagulation, platelet aggregation, and fibrinolysis and intestinal microbiota in thrombosed mice were investigated by ELISA and high-throughput sequencing analysis, respectively.

## 2. Results

### 2.1. Effect of Clam Heparinoids on Anticoagulant Activity In Vitro

As presented in Table 1, anticoagulant potency determined by the Azure A method and anticoagulant time determined by sheep plasma method of clam heparinoid decreased as the molecular weight decreased. The anticoagulant potency of G2 was 157.33 ± 3.87 U/mg, about 74% of that of heparin (HP). The anticoagulation time of physiological saline was 400 ± 25 s (not listed in Table 1). The anticoagulation time of G2 was prolonged by 231% relative to that of saline, approximately 64% of that of HP (360.5%). The anti-IIa of clam heparinoids decreased as the molecular weight decreased, whereas DG1 had the maximum anti-Xa activity, about 90% that of low-molecular-weight heparin (LMWH). The anti-Xa/IIa value of DG1 was 2.13, which is close to that of LMWH [26].

### 2.2. Effect of Clam Heparinoids on Thrombolytic Activity In Vitro

**Figure 1:**
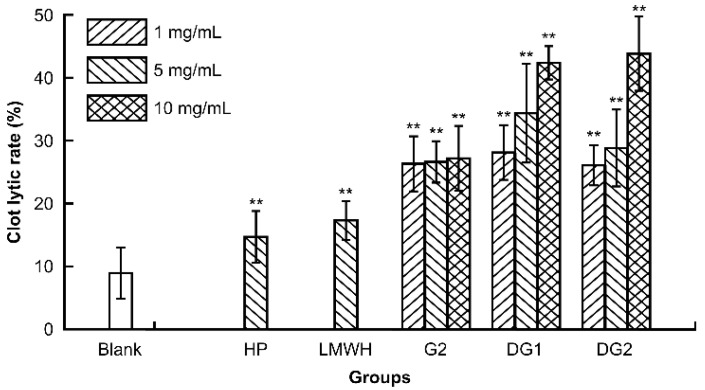
Comparison of the thrombolytic activity of clam heparin with different molecular weights. Statistical significance: * *p* < 0.05, ** *p* < 0.01, as compared with blank group.

A clot lytic rate assay was employed to evaluate the thrombolytic activity in vitro of G2, DG1, and DG2. HP and LMWH were used as references. The clot lytic rate is defined as the ratio of mass of dissolved clot to original weight of clot after thermoregulation of sample solution. As presented in Figure 1, G2, DG1, DG2, HP, and LMWH at the experimental dose significantly enhanced the lytic clot rate in a dose- and molecular-dependent manner. The lytic clot rates of G2, DG1, and DG2 over the experimental dose were markedly more potent than those of HP and LMWH at 5 mg/mL. In addition, the results indicated that G2, DG1, and DG2 had high thrombolytic properties in vitro, which positively correlated with molecular weight.

### 2.3. Effect of Clam Heparinoids on Carrageenan-Induced Venous Thrombosis

**Figure 2:**
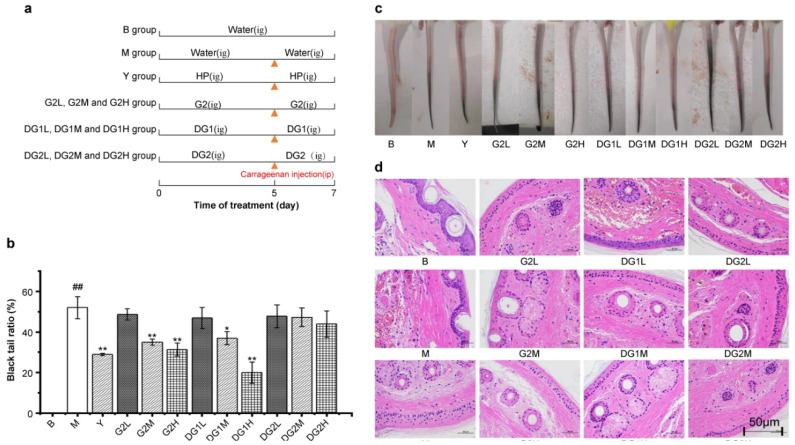
Schematic diagram of the experimental process (**a**); blacktail ratio diagram of the mice in each group (**b**); blacktail images of the mice (**c**); and HE staining section diagram of tail tissue (**d**). B group: the blank control group gavaged with water; M group: the model group gavaged with water and treated with carrageenan; and Y group: the positive control group gavaged with sodium heparin and treated with carrageenan. The suffixes L, M, and H in the group names represent the doses of 10 mg/kg, 20 mg/kg, and 40 mg/kg of gavage samples, respectively. “ig” mean intragastric gavage; “ip” mean intraperitoneal injections. Statistical significance: * *p* < 0.05, ** *p* < 0.01, as compared with model (M) group. ^#^ *p* < 0.05, ^##^ *p* < 0.01, as compared with blank (B) group.

To determine the effect of clam heparinoids on thrombosis, Kunming (KM) mice were treated as indicated in Figure 2a. Samples were gavaged and the caudal thrombus model was established by intraperitoneal injection of carrageenan after gavaging the samples for 5 days and then continuing to gavage the samples for 2 days.

The blacktail ratio, defined as the ratio of the tail length with thrombosis to the whole tail length, reflects the antithrombotic activity of G2, DG1, and DG2. The blacktail ratio of all experimental groups after 48 h of carrageenan injection is presented in Figure 2b. The blacktail ratio of the Y group (group treated by carrageenan and was gavaged with sodium heparin) was significantly lower than that of the M group (group treated by carrageenan but was not gavaged). This suggests that heparin sodium can also inhibit the formation of thrombosis via intragastric gavage, which may be attributed to direct absorption in the intestinal tract [29,30,31,32] or the absorption of intestinal microflora [33]. In addition, G2 at 20 mg/kg and 40 mg/kg and DG1 at 20 mg/kg and 40 mg/kg effectively reduced the blacktail ratio, whereas DG2 demonstrated a negligible capacity to reduce this ratio. The decreasing effect of G2 and DG1 at 20 mg/kg was similar to that of heparin sodium. Compared with the M group, heparin sodium, G2, and DG1 at 20 mg/kg reduced the blacktail ratio by 44.2%, 32.7%, and 24.0%, respectively. Furthermore, the blacktail ratio of DG1 at 40 mg/kg was the lowest. These results indicate that G2 and DG1 interfered with thrombogenesis similar to heparin sodium, suggesting that moderate degradation can maintain clam heparinoid antithrombotic activity.

To further confirm the antithrombotic effects of clam heparinoid treatment, blacktail conditions were photographed, and cross-sections of the tails at similar locations were performed with HE staining. As presented in Figure 2c,d, these results are consistent with the blacktail ratio results and indicate that G2, DG1, and DG2 can alleviate the symptoms of tail thrombosis caused by carrageenan.

### 2.4. Effect of Clam Heparinoids on Fibrinolysis In Vivo

**Figure 3:**
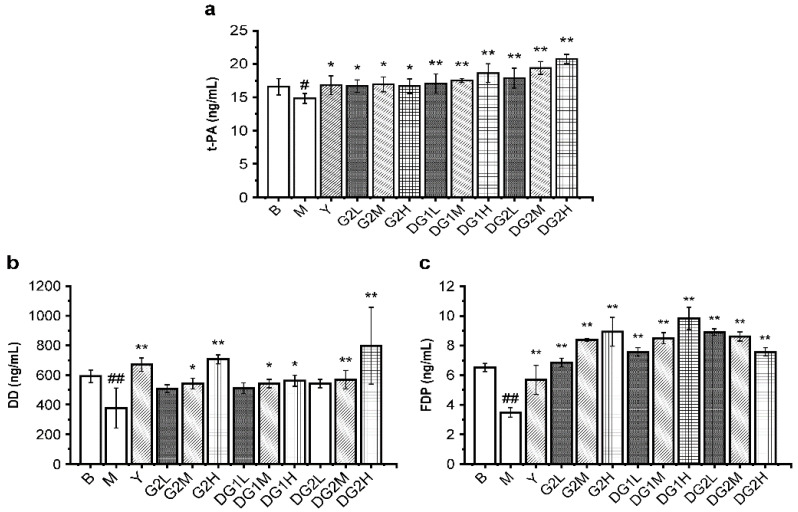
Fibrinolytic indexes in mice of different treatment groups for (**a**) tissue plasminogen activator (t-PA), (**b**) D-dimer (DD) mass concentration, and (**c**) fibrinogen degradation product (FDP). B group: the blank control group gavaged with water; M group: the model group gavaged with water and treated with carrageenan; and Y group: the positive control group gavaged with sodium heparin and treated with carrageenan. The suffixes L, M, and H in the group names represent the doses of 10 mg/kg, 20 mg/kg, and 40 mg/kg of gavage samples, respectively. All experimental groups except the blank control group were injected intraperitoneally with carrageenan. Statistical significance: * *p* < 0.05, ** *p* < 0.01, as compared with model (M) group. ^#^ *p* < 0.05, ^##^ *p* < 0.01, as compared with blank control (B) group.

The fibrinolytic activities in vivo of G2, DG1, and DG2 were also assayed by D-dimer (DD), tissue-type plasminogen activator (t-PA), and fibrin degradation products (FDP). Heparin was used as a positive control. DD, which contains two crosslinked D fragments of the fibrin protein, is produced from crosslinked fibrin by the plasmin’s action. FDP is the degradation product of fibrous protein [34]. t-PA transforms plasminogen into plasmin. As presented in Figure 3, the levels of FDP, DD, and t-PA were significantly enhanced by G2, DG1, and DG2 over the experimental dose range. G2, DG1, and DG2 at 20 mg/kg exerted less of an increasing effect on the DD level and a higher induction of FDP and t-PA than heparin. These results indicate that G2, DG1, and DG2 had high fibrinolytic activity exerted by increasing t-PA, which activated the proenzyme plasminogen into biologically active plasmin, promoted fibrin degradation, and increased the FDP and DD levels.

### 2.5. Effect of Clam Heparinoids on Platelet Aggregation In Vivo

The plasma levels of von Willebrand factor (vWF), 6-keto-prostacyclin-F1α (6-keto-PGF1α), and TXB2 were measured to evaluate platelet aggregation function in vivo. vWF promotes platelet adhesion and aggregation [35]. Thromboxane B2 (TXB2) and 6-keto-PGF1α are also regulators of platelet aggregation. TXB2 promotes platelet aggregation and vasoconstriction, whereas 6-keto-PGF1α has an opposite effect [13].

**Figure 4:**
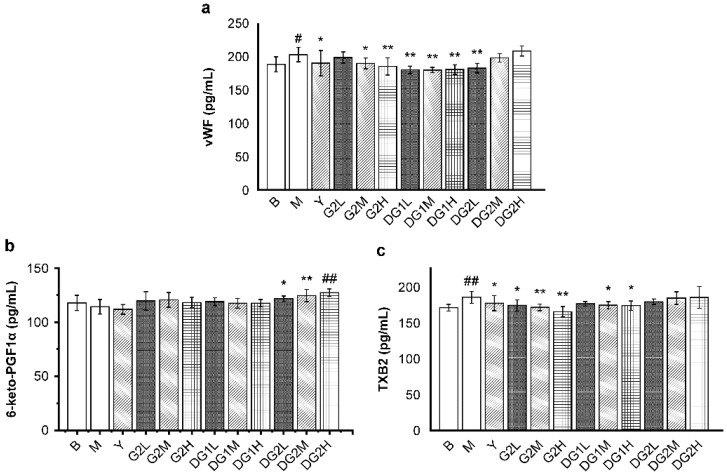
Mass concentrations of platelet aggregation indexes of thromboxane B2 (TXB2) (**a**); 6-keto-prostacyclin-F1α (6-keto-PGF1α) (**b**); and von Willebrand factor (vWF) (**c**) in mice of different treatment groups. B group: the blank control group gavaged with water; M group: the model group gavaged with water and treated with carrageenan; and Y group: the positive control group gavaged with sodium heparin and treated with carrageenan. The suffixes L, M, and H in the group names represent the doses of 10 mg/kg, 20 mg/kg and 40 mg/kg of gavage samples, respectively. All experimental groups except the blank control group were injected intraperitoneally with carrageenan. Statistical significance: * *p* < 0.05, ** *p* < 0.01, as compared with model (M) group. ^#^ *p* < 0.05, ^##^ *p* < 0.01, as compared with blank control (B) group.

As presented in Figure 4a, the vWF level significantly decreased in mice that were treated with heparin sodium at 20 mg/kg; G2 at 20 mg/kg and 40 mg/kg; DG1 at 10 mg/kg, 20 mg/kg, and 40 mg/kg; and DG2 at 10 mg/kg. The enhancing effect of G2 and DG1 on the vWF level was dose-dependent, whereas that of DG2 exhibited a different trend. For the 6-keto-PGF1α levels (Figure 4b), G2 at 10 mg/kg, 20 mg/kg, and 40 mg/kg and DG1 at 10 mg/kg, 20 mg/kg, and 40 mg/kg exerted a slight increasing effect on the 6-keto-PGF1α levels. On the contrary, DG2 at 10 mg/kg, 20 mg/kg, and 40 mg/kg exerted a remarkable increasing effect on the 6-keto-PGF1α levels in a dose-dependent manner. For the TXB2 levels, G2 at 10 mg/kg, 20 mg/kg, and 40 mg/kg and DG1 at 20 mg/kg and 40 mg/kg effectively inhibited TXB2 release in a dose-dependent manner (Figure 4c).

In summary, our results indicate that G2 and DG1, which had antithrombotic effects, mainly exerted antiplatelet aggregation activity by decreasing the vWF and TXB2 plasma levels. DG2 not only increased the level of 6-keto-PGF1α but also increased the TXB2 level.

### 2.6. Effect of Clam Heparinoids on Coagulation In Vivo

**Figure 5:**
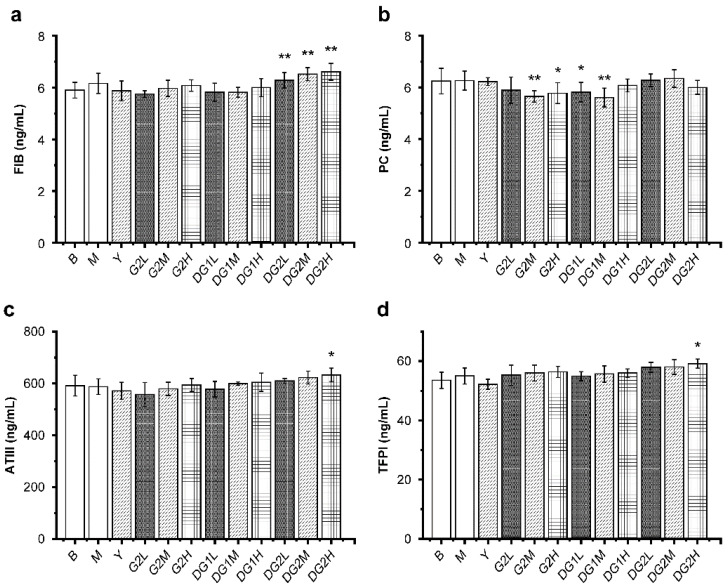
Mass concentrations of the anticoagulation indexes fibrinogen (FIB) (**a**); protein C (PC) (**b**); antithrombin III (ATIII) (**c**); and tissue factor pathway inhibitor (TFPI) (**d**) in mice of different treatment groups. B group: the blank control group gavaged with water; M group: the model group gavaged with water and treated with carrageenan; and Y group: the positive control group gavaged with sodium heparin and treated with carrageenan. The suffixes L, M, and H in the group names represent the doses of 10 mg/kg, 20 mg/kg, and 40 mg/kg of gavage samples, respectively. All experimental groups except the blank control group were injected intraperitoneally with carrageenan. Statistical significance: * *p* < 0.05, ** *p* < 0.01, as compared with model (M) group.

Antithrombin III (ATIII), tissue factor pathway inhibitor (TFPI), and the protein C (PC) system containing PC, protein S, thrombomodulin, and activated PC inhibitor constitute the humoral anticoagulant system [36]. Fibrinogen (FIB) is quickly converted into fibrin, which is an essential component of thrombosis. As presented in Figure 5, compared with the M group, DG2 at 10 mg/kg, 20 mg/kg, and 40 mg/kg increased the level of FIB, and DG2 at 40 mg/kg significantly increased the levels of ATIII and TFPI. Furthermore, the FIB, ATIII, and TFPI levels increased as the doses of G2, DG1, and DG2 increased, and the molecular weight of clam heparinoid decreased. In summary, because the level of PC decreased, the antithrombotic activity of G2 and DG1 cannot be attributed to their capacity to increase the FIB, ATIII, and TFPI levels. DG2, which exhibited negligible antithrombotic activity, exerted an anticoagulation effect by increasing the levels of FIB, ATIII, and TFPI.

### 2.7. Effect of Clam Heparinoids on Fecal Microbiota

**Figure 6:**
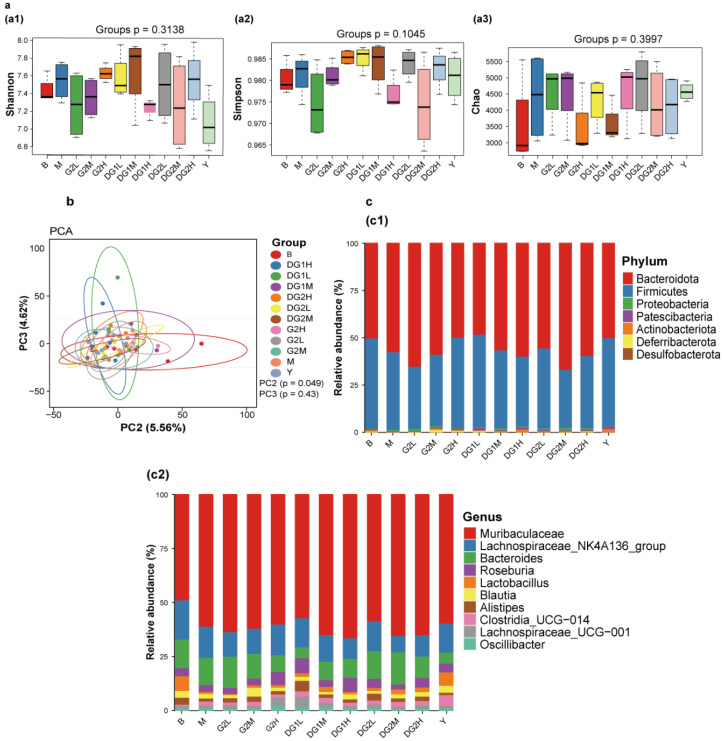
Effects of 7 days of clam heparinoid gavage on the intestinal flora. (**a**) Alpha diversity analysis of the Shannon index diagram (**a1**); Simpson index (**a2**); and ace index diagram (**a3**). (**b**) Beta diversity analysis of the principal component analysis (PCA). (**c**) The intestinal floral composition analysis at the phylum (**c1**) and genus (**c2**) levels. B group: the blank control group gavaged with water; M group: the model group gavaged with water and treated with carrageenan; and Y group: the positive control group gavaged with sodium heparin and treated with carrageenan. The suffixes L, M, and H in the group names represent the doses of 10 mg/kg, 20 mg/kg, and 40 mg/kg of gavage samples, respectively. All experimental groups except the blank control group were injected intraperitoneally with carrageenan.

Figure 6 presents the change in intestinal floral diversity after 7 days of clam heparinoid gavage. The Shannon (Figure 6(a1)), Simpson (Figure 6(a2)), and chaos (Figure 6(a2)) indexes were used to evaluate alpha diversity. The alpha diversity of the M group exhibited no difference from that of the B group. Heparin sodium was able to recover the alpha diversity level to that of the B group. In the G2 group, the Shannon, Simpson, and chaos indexes increased and recovered to the level of the B group. Meanwhile, the Shannon, Simpson, and chaos indexes of the DG1 group showed a different trend and retrieved to the B group at 20 mg/kg. DG2 impacted the alpha diversity in a dose-independent manner, and the DG2L (DG2 at 10 mg/kg by gavage) group demonstrated a similar alpha diversity as that of the B group.

Figure 6b presents the results of the beta diversity of the intestinal flora. Principal component analysis (PCA) revealed the similarities and differences in the intestinal floral composition between different grouped samples. The results indicated that the clam heparinoid group was separated from the M group, especially in the Y (sodium heparin at 20 mg/kg by gavage), G2H (G2 at 40 mg/kg by gavage), DG1L (DG1 at 10 mg/kg by gavage), and DG2L (DG2 at 10 mg/kg by gavage) groups, which were recovered to the level of the B group.

The effects of clam heparinoids on the intestinal floral composition are presented in Figure 6c. Firmicutes and Muribaculaceae were the primary intestinal flora in the B group, but Firmicutes decreased, whereas Muribaculaceae increased in the M group. The effect of clam heparinoids on the intestinal floral composition demonstrated a similar trend as the alpha diversity. The intestinal floral composition of the Y, G2H, DG1L, and DG2L groups recovered to the level of the B group at the phylum and genus levels.

## 3. Discussion

The use of heparinoids with a high molecular weight has been restricted owing to their high hemorrhagic risk and poor solubility and absorption. In previous study, the free-radical degradation method was employed to degrade the large-molecular-weight clam heparinoid G2 (60.25 kDa), yielding the medium-molecular-weight degradation product DG1 (24.48 kDa) and the low-molecular-weight degradation product DG2 (6.75 kDa). The structure of the functional group remains unchanged during degradation, but the higher structure significantly changes [28].

The azure A, sheep plasma, hair color substrate, and clot lytic rate assays were employed to determine the anticoagulant and thrombolytic activities of clam heparinoids in vitro to investigate the effect of molecular weight on their antithrombotic activities. The anticoagulant potency and ability to prolong the anticoagulant time of G2 were approximately 70% that of HP. The anti-Xa/IIa value of DG1 was 2.13, which was close to that of LMWH. DG2 had greater thrombolytic activity than G2, DG1, and heparin. Our findings indicate that all clam heparinoids of varying molecular weights have some degree of antithrombotic potential.

Owing to its high negative charge, considerable molecular weight, and rapid metabolism in the gastrointestinal tract [37], heparin is considered to have no antithrombotic activity by oral administration. However, research suggests that heparin absorbed into the plasma accumulates in the endothelium for several hours and is then released into the plasma, which provides a basis for the oral antithrombotic activity of heparin and LMWH [38,39]. In addition, degradation can improve oral bioavailability. Hiebert et al. reported that orally administered LMWHs, logiparin (tinzaparin) and reviparin, have antithrombotic activity at much lower doses than UFHs in the same model [40,41]. Zhao et al. discovered that oral administration of the MMW fucoidan from *Laminaria japonica* exerted lower antithrombotic activity than the LMW fucoidan [42]. In this study, we observed that oral administration of the low-molecular-weight clam heparinoid DG2 exerted negligible antithrombotic activity. The high-molecular-weight clam heparinoid G2 at 10 mg/kg and 20 mg/kg and the medium-molecular-weight clam heparinoid DG1 at 10 mg/kg and 20 mg/kg exhibited similar antithrombotic activity. In comparison, DG1 at 40 mg/kg had a higher muscular antithrombotic activity than G2. Thus, our results suggest that moderate degradation improves oral bioavailability, whereas excessive degradation has a negative effect on antithrombotic activity. In addition, the structure of G2 was different from those exerted by mammals [27], and DG2 exhibited weak anticoagulant, anti-Xa, and anti-IIa activities in vitro, which may be the reason for the negligible antithrombotic activity of DG2.

To reveal the mechanisms of action on the antithrombotic activity of clam heparinoids by oral administration their effects on coagulation, platelet aggregation, and fibrinolysis in thrombotic mice were examined. In our study, we observed that oral administration of G2 and DG1 for 7 days exerted excellent fibrinolytic and moderate antiplatelet activities by promoting t-PA, FDP, and DD and downregulating TXB2 and vWF, which was consistent with the effects of heparin sodium. In previous studies, G2 exerted moderate fibrinolytic activity by promoting t-PA and downregulating PAI-1. Thus, this is the first report of oral administration of clam heparinoids promoting antiplatelet activity by downregulating the TXB2 and vWF levels in mice. However, it is important to study whether this effect is induced by genetic regulation or the promotion of existing factors.

The relationship between heparin and gut flora is another vital actor involved in the antithrombotic activity and bioavailability of oral heparin. Zhou et al. reported that orally administrated heparin was mainly distributed in the gastrointestinal tract of mice and was structurally stable in simulated gastric and intestinal fluids in vitro [43]. Our results indicate that clam heparinoids regulate the structure of intestinal flora, which is consistent with the results of Shang et al. [44] and Zhu et al. [45]. However, clam heparinoids had no specific function to promote beneficial bacteria, inhibit harmful bacteria, and regulate diversity, which may be caused by host specificity, model type, amount and type of polysaccharide, and interaction time [44,46,47,48]. Therefore, further studies should be conducted to determine the interaction of clam heparinoids with intestinal flora.

## 4. Materials and Methods

### 4.1. Materials

Fresh clams (*C. antiquata*) were sampled from Zhanjiang, China. Clam heparin G2, DG1, and DG2 were extracted and prepared according to our previous report [28].

Heparin (2111 IU/10 mg) and LMWH (anti-Xa 149.7 IU/mg, anti-IIa 57.1 IU/mg) were products of the National Institutes for Food and Drug Control (Beijing, China). Rat ATIII, PC, FIB, TFPI vWF TXB2, 6-keto-PGF1α, t-PA, DD, and FDP ELISA kits were purchased from Shanghai Langton Biotechnology Co., Ltd. (Shanghai, China). The other chemicals were of analytical grade.

Male KM mice (20–22 g) were provided by Zhuhai Bestest Biotech Co., Ltd. (Zhuhai, China). The animals were acclimated for at least 1 week at a temperature of 24 °C ± 1 °C and a humidity of 55 % ± 5 %. The study protocol was approved by the ethics committee of the Guangdong Ocean University (GDOU-LAE-2021-003) (Guangdong, China).

### 4.2. Assay of Anticoagulant Activity In Vitro

The in vitro anticoagulant potency of G2, DG1, and DG2 was measured using the azure A method. Different volumes of 2 U/mL heparin (0.2 mL, 0.4 mL, 0.6 mL, 0.8 mL, and 1.0 mL) and distilled water were mixed to make a final volume of 2.0 mL. Next, 2.0 mL of 0.05 mol/mL of barbiturate buffer (pH = 8.6) and 0.5 mL of 0.2 mg/mL azure A solution were added. The solution was mixed well and allowed to stand for 5 min, and the UV absorbance (505 nm) was measured. A standard curve was prepared via the UV absorbance of different active heparin concentrations (0.4 U/mL, 0.8 U/mL, 1.2 U/mL, 1.6 U/mL, and 2.0 U/mL). In addition, 0.2 mL of 0.05 mg/mL G2, DG1, and DG2 were mixed with 1.8 mL distilled water, 2.0 mL of 0.05 mol/mL barbiturate buffer (pH = 8.6), and 0.5 mL of 0.2 mg/mL azure A solution. After resting for 5 min, their UV absorbance at 505 nm (double-beam UV–vis spectrophotometer TU-1901; Beijing General Instrument Co. Ltd., Beijing, China) was measured, and the anticoagulant potencies of G2, DG1, and DG2 were quantitatively calculated based on the UV absorbance of the solution [48].

The sheep plasma method was employed to measure the anticoagulant time of G2, DG1, and DG2. G2, DG1, and DG2 (1 mg/mL each) were prepared with saline, with saline alone as the negative control, and heparin was utilized as the positive control. Sheep plasma (100 µL), sample solution (20 µL), and 0. 025 mol/L calcium chloride solution (100 µL) were added to a 2 mL centrifuge tube. The centrifuge tube was inverted three times and placed in a 37 °C water bath. The tube was gently inverted every 30 s, and the clotting time was recorded until the blood stopped flowing [49].

Incubations were performed in 96-well plates. The final concentrations of the reactants were 10 nM of antithrombin, 2 nM of thrombin or factor Xa, and 0–100 µg/mL of sulfated glycans in 40 µL of TS/PEG buffer (0.02-M Tris/HCl, 0.15-M NaCl and 1.0 mg/mL polyethylene glycol 8000, pH 7.4). Thrombin or factor Xa was added last to initiate the reaction. After incubation for 60 s at 37 °C, 25 µL of the chromogenic substrate (0.4 mM) S-2238 for thrombin or S-2222 for factor Xa (Chromogenix AB, Mondal, Sweden) was added, and the absorbance at 405 nm was recorded (Varioskan Flash 2.4; Thermo Fisher Scientific, Waltham, MA, USA). The rate of change of absorbance was proportional to the remaining amount of thrombin or factor Xa activity. Thrombin activity was defined as 100 % in control samples lacking clam heparinoid [50,51].

### 4.3. Assay of Thrombolytic Activity In Vitro

The in vitro thrombolytic activities of G2, DG1, and DG2 were measured using a clot lytic rate assay. Blood was drawn from the eyeball veins of KM mice and placed at room temperature until a big blood clot formed. The clot was washed with saline solution and the liquid on the surface of the clot was removed using filter paper. The clot was cut into pieces, weighed, and placed into polyethylene tubes. The tubes were randomly divided into eight groups (12 clots/group): G2, DG1, and DG2 (1 mg/mL, 5 mg/mL, and 10 mg/mL), heparin, LMWH (5 mg/mL), and saline solution groups. A sample (1 mL) was added to the tube and incubated for 24 h at 37 °C. The residual clot was removed, as well as the liquid on the surface of the clot. The wet weight of the residual clot was measured. The lytic clot rate was calculated using the following equation: lytic clot rate (%) = (1 − Wr/ W) × 100, where W denotes the wet weight of the whole clot, and Wr denotes the wet weight of the residual clot [52].

### 4.4. Effect on Carrageenan-Induced Venous Thrombosis

The experimental mice were randomly divided into 12 groups (*n* = 12): the blank group (B); heparin group (Y); model group (M); low-, medium-, and high-dose G2 groups (G2L, G2M, and G2H, respectively); low-, medium-, and high-dose DG1 groups (DG1L, DG1M, and DG1H); and low-, medium-, and high-dose DG2 groups (DG2L, DG2M, and DG2H). The low-, medium-, and high-dose groups of clam heparinoids were given 10 mg/kg, 20 mg/kg, and 40 mg/kg of clam heparinoids daily by gavage for 7 days, respectively. The mice in the B and M groups were gavaged with an equal volume of normal saline. The mice in the Y group were given 20 mg/kg of heparin gavage. All groups except the B group were intraperitoneally injected with 50 mg/kg of carrageenan on day 5, whereas the mice in the B group were injected with normal saline in the same way. Mice blacktails were observed 24 and 48 h after modeling, and the entire tail length and thrombosed blacktail length were measured using vernier calipers. Then, we calculated the blacktail ratio of the mice. The blacktail ratio = (A1/A2) × 100%, where A1 is the blacktail length and A2 is the entire length of the tail. Two days after carrageenan injection, all mice were anesthetized and euthanized, and then blood and tail samples were collected. All study procedures are performed as per principles described in the Declaration of Helsinki.

### 4.5. Histopathologic Analysis

After the experiment, the tails were photographed. A piece of the tail at the same location (defined as the distance from the tail tip) was collected and then fixed in 4% paraformaldehyde overnight. The samples were then incubated in a 30% sucrose solution overnight, followed by embedding in the OCT solution. Frozen sections (5-µm) were prepared from tissue samples and processed as follows: the sections were incubated in phosphate-buffered saline for 30 min and then stained for nucleus and cytoplasm with hematoxylin solution for 1 min and eosin solution for 5 min. After adequate drying, the slides were observed under a Leica DM3000 microscope (Wetzlar, Germany), and images were taken.

### 4.6. Measurement of Coagulation, Platelet Aggregation, and Fibrinolysis Parameters

The mice were anesthetized and sacrificed. Whole blood samples were centrifuged at 4 °C for 10 min at 3000 r/min to collect the plasma. The plasma levels of TF, TFPI, vWF, TXB2, 6- keto-PGF1α, t-PA, DD, and FDP were evaluated according to the manufacturer’s instructions.

### 4.7. Fecal Microbiota Analysis

After gavage on day 7, fresh feces were collected within 1 h. Four samples were collected from each group, promptly frozen by liquid nitrogen, and stored at—80 °C. Nucleic acid extraction or purification reagent (Hangzhou Guhe: GHFDE100) was used for DNA extraction. The primers used in PCR were the V3–V4 universal primers for the Illumina sequencing platform: forward 5′-CCTACGGGNGGCWGCAG-3′ and reverse 5′-GACTACHVGGGTATCTAATCC-3′. The PCR mixture contained 25 µL of PCR Mix Buffer, 3 µL of dimethyl sulfoxide, 3 µL of forward primer (1 µM), 3 µL of reverse primer (1 µM), 10 µL of purified DNA (5 ng/µL), and nuclease-free water to a final volume of 50 µL. The PCR cycling conditions included an initial denaturation for 30 s at 98 °C, 30 cycles of 15 s at 98 °C, 15 s at 58 °C, and 15 s at 72 °C and a final extension for 1 min at 72 °C. The PCR products were purified using AMPure XP Beads (Beckman Coulter, Indianapolis, IN, USA) and library quantification was performed using Qubit. After quantification, the libraries were sequenced using the Illumina NovaSeq platform (Illumina, San Diego, CA, USA). Vsearch v.2.15.0 software was used to match the raw data, and the Quality Control software package was used for filtering. The criteria for filtering low-quality sequences were as follows: sequences of less than 150 bp long, average quality of less than 20, sequences containing unclear bases, and sequences containing single nucleotide repeats >8 bp long. The filtered clean reads were clustered to obtain ASV statistics, and representative sequences of operational taxonomic units were selected using default parameters. The representative sequences are annotated with species by qiime2 based on the silva138 database. All sequences were classified by operational taxonomic unit at a similarity level of 97%, and bioinformatics analysis was conducted [53].

### 4.8. Statistical Analysis

The results are expressed as mean ± standard deviation. The experimental data were subjected to analysis of variance for a completely random design, and three samples were prepared for each assay. Data processing was performed using SPSS 17 and Origin 8.

## 5. Conclusions

Our results demonstrate that the molecular weight of heparin impacts its antithrombotic activity. Furthermore, molecular weight is an essential chemical characteristic for clam heparinoids administered by the oral routine. Further, DG1 had better absorption and antithrombotic activities than G2. Considering the large molecular weight of G2, the medium-molecular-weight clam heparinoid DG1 seems to be an attractive choice as an oral antithrombotic agent to prevent thrombotic disease.

## Figures and Tables

**Table 1:** Effects of clam heparinoids on anticoagulant activity in vitro.

Compounds	Molecular Weight (kDa)	Anticoagulant Potency (U/mg)	Anticoagulant Time (s)	Anti-Xa Activity (UI/mg) ^[ref]^	Anti-IIa Activity (UI/mg) ^[ref]^	Anti-Xa/IIa ^[ref]^
HP ^a^	-	211.1	1842 ± 16	-	-	-
LMWH ^a^	-	-	-	149.7	57.1	2.62
G2	60.25	157.33 ± 3.87	1324 ± 12	102.93	89.49	1.15
DG1	24.48	48.43 ± 2.65	714 ± 45	137.26	64.34	2.13
DG2	6.75	26.86 ± 1.62	515 ± 25	36.95	38.37	0.96

^a^ HP: heparin; LMWH: low-molecular-weight heparin; both HP and LMWH are standards specifically designed for potency determination. ^[ref]^: refer to previously obtained data.

## Data Availability

The data presented in this study are available on request from the corresponding author. The data are not publicly available due to public availability violating the consent that was given by research participants.
